# MED12-STAT1-TAP2 axis regulates CD8 + T cell cytotoxicity and mediates immunotherapy outcome in non-small cell lung cancer

**DOI:** 10.1007/s10142-025-01690-2

**Published:** 2025-09-01

**Authors:** Minghao Feng, Yuxu Niu, Jiayuan Liu, Gang Liu

**Affiliations:** 1 Department of Thoracic Surgery, Shanghai East Hospital, Tongji University, 1800 Yuntai Road, Shanghai, 200120 China; 2 Department of Thoracic, The Sixth People’s Hospital, Shanghai Jiaotong University, No.600, Yishan Rd, Shanghai, 200233, 210508 PR China

**Keywords:** NSCLC, MED12, Immunotherapy, Prognosis, Micro-environment

## Abstract

Although immunotherapy for late-stage non-small cell lung carcinoma (NSCLC) has been clinically utilized, its prognosis remains highly heterogeneous, prompting us to investigate novel predictive immunotherapy biomarkers for NSCLC. We analyzed the correlations between MED12 nonsynonymous mutations and survival, clinical, genomic, transcriptomic information, and immune infiltration information through data mining across multiple datasets. We also investigated the mechanism of MED12 using luciferase assay, Western blot, ChIP-PCR, and siRNA. MED12 is significantly associated with survival in completely independent immunotherapy datasets, including MSKCC (*N* = 350), Naiyer2015 (*N* = 34), our own (*N* = 295) and the pan-cancer dataset, but not in the TCGA dataset, where patients received non-immunotherapy regimens. Mutations in MED12 showed no significant correlation with known metrics (TMB, IPS/CTLA4/PD1 status, PD-1/PD-L1 expression, and TCR/BCR status) or DNA Damage Repair (DDR) pathway mutations, yet they carried independent prognostic information according to the Cox multivariate regression. On the other hand, MED12 mutation is significantly associated with multiple immune-related pathways and immune infiltration of CD8 + T cells and activated NK cells. Lactate dehydrogenase assay revealed that knockdown of TAP2 restored the upregulation of CD8 + T cell cytotoxicity triggered by MED12 knockdown. ChIP-PCR, luciferase assay and siRNA knock down assay indicate that MED12 binds to the promoter region of STAT1 to suppress its transcription, while the transcription factor STAT1 promotes the transcription of TAP2, thus inhibiting the antigen processing and presentation. Collectively, MED12 mutation is an independent and valuable biomarker for predicting the response to immune checkpoint inhibitor (ICI)therapy in NSCLC by modulating CD8 + T cell cytotoxicity via the STAT1/TAP2 axis.

**Supplementary Information:**

The online version contains supplementary material available at 10.1007/s10142-025-01690-2.

## Introduction

It was estimated that there were 2,480,301 new lung cancer cases in 2022, accounting for 12.4% of all cancer cases and causing 18.7% of cancer-related deaths(Bray et al. 2024; Siegel et al. 2022). Among the lung cancer patients, ~ 85% were diagnosed with non-small cell lung cancer (NSCLC), according to the World Health Organization (WHO)(Travis et al. 2015). Recently, immunotherapy, especially drugs targeting PD-1/PD-L1/CTLA4, has ben emphasized due to the favorable objective response rate (ORR), especially for late-stage or multi-line treated patients(Reck et al. 2022). For example, in advanced NSCLC, the ORR of patients treated with nivolumab plus ipilimumab was 38%, with a progression-free survival 2 (PFS2) of 13.9 months, which was higher than that of the control group in the Checkmate 9LA trial (Reck et al. 2021). However, a high proportion of NSCLC patients could not benefit from the immunotherapy. Therefore, predictive and prognostic biomarkers remain a challenge for ​current research.

In the last few years, prognostic and predictive biomarkers have been reported for NSCLC immunotherapy. For example, PD-L1 has been widely reported as an effective biomarker. In a clinical trial that enrolled 563 NSCLC patients with at least 50% PD-L1 + patients, the median progression-free survival (PFS) time of the cemiplimab monotherapy group was 8.2 months, which was significantly longer than that of the chemotherapy group(Sezer et al. 2021). Similarly, for metastatic NSCLC, the median overall survival time of the atezolizumab treatment group reached 20.2 months (HR = 0.59) with the highest PD-L1 expression(Herbst et al. 2020). Tumor mutation burden (TMB), consisting of blood TMB (bTMB) and tissue TMB (tTMB), has been reported as a valuable biomarker for immunotherapy. Marcin et al. reported that TMB levels in 567 NSCLC patients was significantly correlated with anti-PD-L1/PD-1 efficacy in a clinical trial(Kowanetz et al. 2017). Similarly, in KEYNOTE-101 trial (pembro monotherapy), tTMB was shown to be correlated with overall survival (OS), progression-free survival (PFS) and ORR(Herbst et al. 2019). Furthermore, TMB was approved by the FDA as a biomarker for unresectable or metastatic solid tumors treated with pembrolizumab(Marcus et al. 2021). Single-gene mutation was also reported for its role in predicting the treatment response or prognosis in NSCLC. As a frequent mutation, KRAS G12D was reported to repress the immune response and reduce the sensitivity of NSCLC to the efficacy of immune checkpoint inhibitors (ICIs)(Hsu et al. 2022). Another study analyzed mutations of PTPRD/PTPRT in NSCLC and revealed that these mutations were significantly associated with PFS(Wang et al. 2021). Similarly, EPHA5 was shown to be associated with durable clinical benefit and survival of NSCLC patients. Nevertheless, the mutation frequency of these genes (except for genes like TP53) is relatively low. Thus, screening more predictive and prognostic biomarkers for NSCLC is stillan area requiring further investigation.

Herein, by analyzing publicly available databases, we identified MED12 (mediator complex subunit 12) mutations as a prognostic and predictive biomarker for ICI. NSCLC samples harboring this mutation have prolonged survival and better clinical response across datasets. Furthermore, clinical, genomic and transcriptomic analyses demonstrated that MED12 mutations are associated with multiple biological features of NSCLC, particularly ​immune-response-related pathways. In addition, we revealed that MED12 mutation enhances the expression of STAT1 and thus leading to elevated TAP2 expression, thereby enhancing CD8 + T cell cytotoxicity.

## Material and method

### Sample enrollment and data collection

The MSKCC-NSCLC dataset was retrieved from cBioPortal database (https://www.cbioportal.org/)(Cerami et al. 2012), and the processed mutational and clinical information was retrieved. Only samples clearly labeled as NSCLC were retained for further analyses, resulting in 350 enrolled samples. In TCGA-NSCLC dataset, mutation, gene expression and clinical information were also downloaded from cBioportal. In this dataset, primary NSCLC samples with mutational information were retained, and 997 samples were enrolled. In addition, mutational, survival, and clinical information of Naiyer 2015 (Rizvi et al. 2015) and Hira 2018 (Rizvi et al. 2018) datasets were also downloaded from the supplementary files provided in the corresponding articles. There were 34 and 240 samples enrolled in Naiyer 2015 and Hira 2018 datasets. Since the data were retrospectively collected, some some clinical information may be missing.

### Mutational analyses and DDR pathways

The mutation information of MSKCC-NSCLC dataset was generated from gene panel-based sequencing while TCGA-NSCLC dataset was generated using whole exome sequencing (WES). Thus, the co-mutation and exclusive mutation analyses of MSKCC-NSCLC and TCGA-NSCLC datasets were performed separately. Fisher’s exact test was used for co-/exclusive mutational gene identification, and R package ‘GenVisR’(Skidmore et al. 2016) was used for visualization of the mutational landscape. DNA Damage Repair (DDR) pathways and the involved genes in all pathways were retrieved from a previous study. A pathway was considered mutated ​if at least one gene within it harbored a non-synonymous mutation. The relationship between mutations in MED12 and DDR pathways was also assessed using Fisher’s exact test, and p < 0.05 was considered statistically significant.

### GO/KEGG/GSEA analyses and immune infiltration evaluation

Differentially expressed genes were identified in the TCGA-NSCLC dataset using R package ‘limma’(Ritchie et al. 2015) with adjusted p-value < 0.05 and log2-fold change<−0.5 or > 0.5. The GO/KEGG enrichment analyses were performed and visualized with R packages ‘enrichplot’ and ‘clusterProfiler’(Yu et al. 2012). Gene Set Enrichment Analysis (GSEA) was carried out with the a ranked list based on p-values and log2-fold changes generated from ‘limma’, using the R package ‘GSEAbase’.

Immune infiltration abundances were estimated using different algorithms, including CIBERSORT(Newman et al. 2015), EPIC(Racle et al. 2017), TIMER(Li et al. 2020), QUANTISEQ(Finotello et al. 2019) and XCELL(Aran et al. 2017). Differences in immune cell infiltration between MED12-wild type and MED12-mutated groups were identified and visualized with R packages ‘ComplexHeatmap’ and ‘vioplot’.

### Luciferase assay and LDH assay

Cells were seeded into a 24-well plate with 400 µL of medium. 1 µL of TSnanofect V1 was mixed with 49 µL of serum-free medium, and then this mixture was mixed with another 50 µL of TSnanofect V1. After incubation for 5 min, 100 µL of the transfection solution was added, and the volume was supplemented to 500 µL. The cells were cultured at 37 °C for 48 h. Subsequently, the medium was removed and 100 µL of PLB lysis solution was added. After shaking for 15 min, 20 µL of the cell lysate was added. After the firefly luciferase intensity was quantified, 100 µL of Stop & Glo^®^ reagent was added, and the Renilla luciferase signal was also determined.

Cytotoxicity of CD8 + T cells were determined using the Lactate Dehydrogenase (LDH) Cytotoxicity Assay kit (Beyotime Institute of Biotechnology) according to the manual provided. Human CD8 + T cells (IMP-H175, Immocell) was cultured withh NCI-1299 or A549 cell lines at 37℃ for 4 h. The LDH levels were determined using normalized OD490 values.

### ChIP assay

SimpleChIP@ Enzymatic Chromatin IP kit (9002 S, Cell Signaling Technology) was used for ChIP according to the manual provided. A549 and NCI-1299 cells were cultured to a density of ~ 1 × 10⁷ cells, and treated with 1% formaldehyde. The chromatin was collected and digested with micrococcal nuclease. Subsequently, the nuclear membrane was disrupted, and the DNA was fragmented into about 150–900 bp. The samples were immunoprecipitated with IgG, MED12 (20028-1-AP, proteintech, Wuhan), or STAT1 (10144-2-AP, proteintech, Wuhan) antibody. After washing, reverse cross-linking, and elution, the PCR was used for quantitative evaluation.

### Western blot (WB) assay

Samples were added to separation gel, electrophoresis was performed, and the gel was washed. The membrane was transferred at 80 V for 100 min in ice-water. The PVDF membrane was stained with Ponceau S and washed. The membrane was blocked for 2 h and washed with 1×TBST elution solution for 2 min. The membrane was incubated with the primary antibody while shaking at 4 °C overnight. Then, the membrane was incubated with secondary antibody for 1.5 h. The membrane was washed with 1×TBST three times, 15 min each. The membrane was transferred to a chemiluminescence instrument, supplemented with the luminescence solution and photographed under microscope. The antibodies are listed as follows: anti-MED12 (20028-1-AP, proteintech, Wuhan), anti-STAT1 (10144-2-AP, proteintech, Wuhan), anti-TAP2 (10161-1-AP, proteintech, Wuhan).

### RNA extraction and qRT-PCR assay

Cells were lysed and centrifuged. 150 µL of Dilute buffer was added to the sample and the mixture was vortexed, followed by incubation for 5 min. The sample was centrifuged at 12,000 g for 15 min, 500 µL of the upper layer was extracted, and 500 µL of pre-cooled isopropanol was added. The sample was shaken, allowed to stand for 15 min and centrifuged at 12,000 g for 15 min at 4 °C. Discard the supernatant, add 1 mL of pre-cooled 75% ethanol to the sample, and then centrifuge the sample. Discard the supernatant, wash the sample with 75% ethanol, and dry it at 37 °C for 1–3 min. Dilute the sample to about 500 ng/µL, and quantify its concentration. Perform electrophoresis at 120 V for 25 min to generate ~ 1 µg of total RNA. Perform reverse transcription to generate cDNA, and then record the expression levels of reference and target genes using a qPCR instrument. Normalize the gene expression values using endogenous reference. The primer sequences are listed as follows: MED12-F: GGGATCTTGAGCTACGAACAC, MED12-R: GCAGGCTGGTTATTGAAACCTTG, TAP2-F: AATCCCTCACTATTCTGGTCGT, TAP2-R: TCGAGACATGGTGTAGGTGAAG, STAT1-F: CAGCTTGACTCAAAATTCCTGGA, STAT1-R: TGAAGATTACGCTTGCTTTTCCT.

### Overexpression and SiRNA experiment

All cell lines involved were obtained from the National Collection of Authenticated Cell Cultures in China (www.cellbank.org.cn), and the STR reports were also obtained accordingly. Lentivirus overexpressing TAP2 and STAT1 were purchased from GenePharma (Shanghai, China). The cells were transfected with 120 pmol siRNA duplexes using the standard protocol and incubated for 72–96 h. The siRNA sequence was as follows: siMED12-1:UUCUCUGCAAUAAUGCUGCUG, siMED12-2:UAUUACAACGUAAUUUCUCUG, siTAP2-1:UUUGUUGUUCACCUGGUCCUG, siTAP2-2:UAGCUGUUACCAAUGUCGCUA, siSTAT1-1:UUAUCCUGGAGAUUACGCUUGCUUU, siSTAT1-2:UUGUCUGUGGUCUGAAGUCUA.

### Statistics

All statistical analyses in this study were conducted using R (v4.3.0) and relevant packages. Survival analyses and Cox proportional hazards regression were performed with the survival package. For continuous variables, Student’s t-test was used if group variances were equal; otherwise, the Wilcoxon rank-sum test was applied. For categorical variables, the Fisher exact test was employed. During Cox multivariate regression, age, sex and pathological stages (if available) was used for adjusted variables in all survival analyses.

## Results

### MED12 mutation predicted the survival of NSCLC

**Fig. 1:**
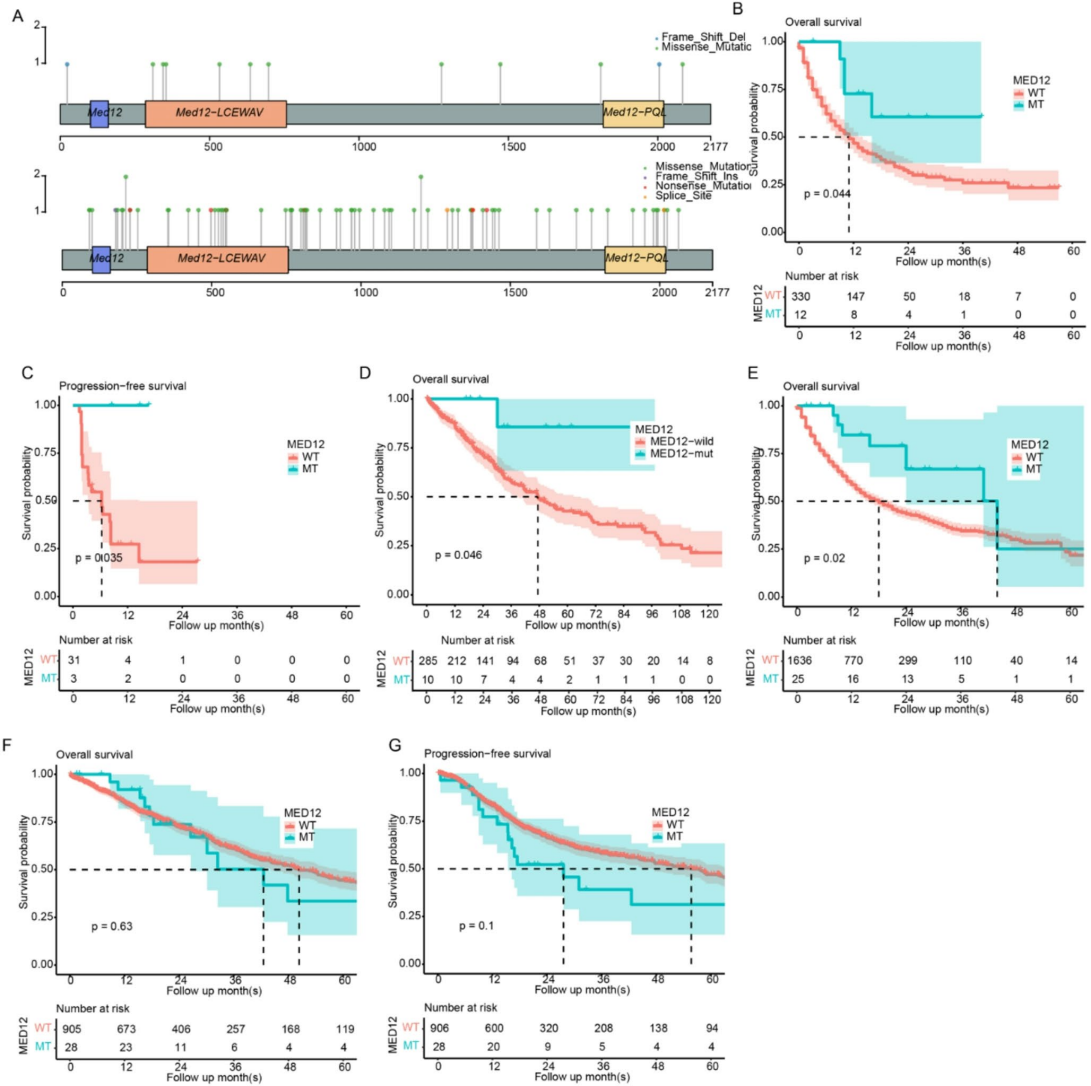
MED12 Mutation Predicted the Improved Survival of NSCLC​. **a**, the distribution of MED12 mutation sites across the coding region in TCGA and MSKCC datasets; **b**, the comparison of overall survival between MED12-mut and MED12-wild-type groups in the MSKCC dataset; **c**, the survival comparison in Naiyer2015 NSCLC cohorts treated with ICIs, with MED12-mutated and wild-type samples; **d**, survival comparison of MED12-wild type and mutated samples in our own dataset; **e**, the pan-cancer survival analysis using samples from 11 cancer types in the MSKCC dataset; **f**-**g**, the overall and progression-free survival analyses in the TCGA - NSCLC dataset

The datasets used for genomic analyses are summarized in Table 1. First, we analyzed the mutation pattern of MED12 in TCGA and MSKCC datasets. Samples harboring these non-synonymous mutations were identified as MED12-mutant (MED12-mut) samples, while the others were MED12-wild-type samples. The mutation rates of MED12 in TCGA and MSKCC datasets were 3.42% (12/350 samples) and 3.31% (31/997 samples), respectively. These mutation sites in TCGA dataset were distributed sparsely across the coding region (Fig. 1a). 50% of the mutation sites in MSKCC dataset were located in LCEWAV region of MED12, which is a conserved sequence motif(Lu et al. 2020). Previous study indicated that this region was crucial for CDK8-mediatior complex formation(Banaganapalli et al. 2016). In addition, most mutation types are missense mutation (10/12 in MSKCC). We visualized the missense mutation sites in MSKCC dataset in 3D structure (Fig. S1). The survival of MED12-mut and MED12-wild-type groups was compared in the MSKCC dataset. As a result, the MED12-mut group had significantly better overall survival compared to the MED12-wild-type group (Fig. 1b, *p* < 0.05). To validate this finding, an additional NSCLC cohorts treated with ICIs, Naiyer2015 (*N* = 34), was used for validation. Despite the limited number of MED12-mutated samples (only three samples were identified as the MED12-mut samples in both Naiyer2015), MED12-mutated NSCLC samples showed a significantly prolonged survival period compared to the wild-type group (Fig. 1c, Table S1), consistent with the MSKCC dataset. In addition to the public dataset, we also retrospectively collected MED12 mutation information from 295 patients who received immunotherapy from our affiliation. Consistent with previous result, the survival of MED12-mutated patients has significantly prolonged survival time compared to the wild type (Fig. 1d, *p* = 0.046, Table S1). Furthermore, pan-cancer analyses using all samples in the MSKCC dataset originating from 11 cancer types (*N* = 1661) also revealed that MED12-mutated cancers had a significantly longer survival period (Fig. 1e, Table S1). The adjusted survival curve showed a similar pattern (Fig. S2). In contrast, most samples in the TCGA-NSCLC dataset were not treated with ICIs, and no significant overall or progression-free survival difference was detected (Fig. 1f-g, Table S1). MED12 mutation not only correlated with the survival of ICI-treated NSCLC patients but also associated with pathological response. Analyses of the Naiyer2015 dataset revealed that MED12 mutated samples tended to have a partial response while only progression of disease or stable disease was observed (Table 2, *p* < 0.05, Fisher’s exact test), despite that it is descriptive only. These results indicate that MED12 mutation is a specific biomarker for NSCLC treated with ICIs, suggesting that MED12-mutant NSCLC patients benefit from ICI therapy.

**Table 1:** Detailed information of datasets used in this work

		MSKCC			TCGA			Naiyer2015			Ours		
		WT	MT	pval	WT	MT	pval	WT	MT	pval	WT	MT	pval
Age	< 60	106	3	0.38	211	5	0.65	14	1	1	45	6	0.07
	> 60	238	3		680	23		17	2		240	4	
Gender	Female	175	5	0.21	358	24	1.10E-06	16	2	1	83	4	0.48
	Male	169	1		559	4		15	1		202	6	
Stage	I				467	16	0.51						
	II				258	9							
	III				158	2							
	IV				31	1							
TMB		9.7 ± 9.9	12.5 ± 6.7	0.19	9.6 ± 9.0	15.2 ± 13.3	0.02						
PD-L1					6.2 ± 1.7	6.6 ± 1.9	0.31						

**Table 2:** MED12 mutation status and response (Fisher’s exact test). Descriptive trend observed (*p* = 0.038), but limited by small mutant sample size (*n* = 3)

	MT	WT	*p* = 0.038
PR	3	9	
POD/SD	0	22	

PR, partial response; SD, stable disease; POD, progression of disease

### MED12 mutation is independent of clinical and genomic features

**Fig. 2:**
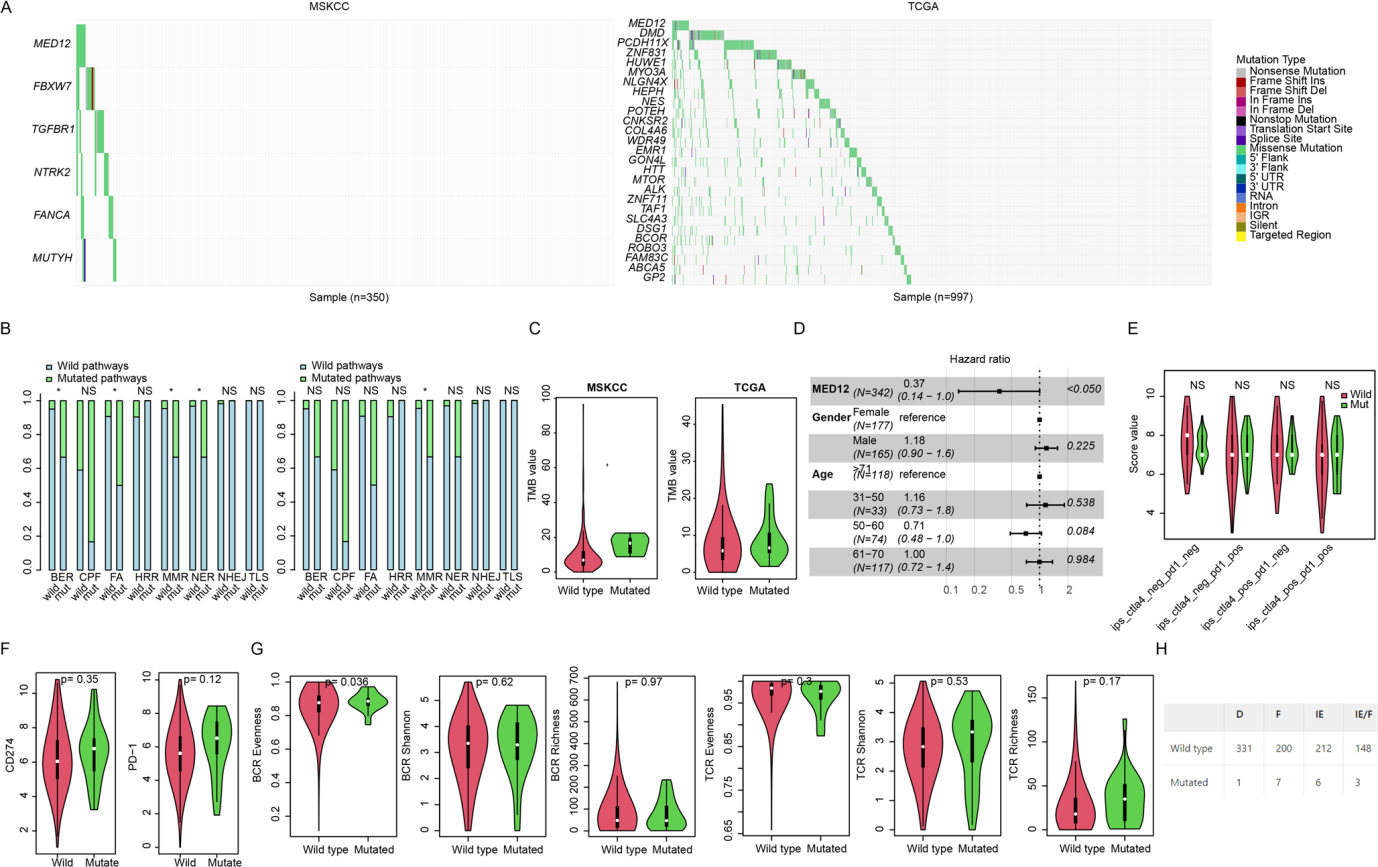
MED12 mutation is independent of clinical and genomic features​. **a**, Genes co-mutated with MED12 in the MSKCC and TCGA datasets; **b**, the relationship between MED12 mutation and DNA damage repair (DDR) pathways; **c**, the evaluation of the correlation between MED12 mutation and tumor mutational burden (TMB) in the MSKCC and TCGA datasets (Naiyer2015 and Hira2018 excluded due to limited MED12-mutant samples); **d**, the result of Cox multivariate regression in the MSKCC dataset; **e**, the relationship between MED12 mutation and IPS CTLA4/PD1 status; **f**, Association between MED12 mutation and PD-1/PD-L1 gene expression; **g**, the relationship between MED12 mutation and BCR and TCR clonality; **h**, the relationship between MED12 mutation and immune subtypes

We investigated the relationship between MED12 mutation and genomic features. Co-mutated genes of MED12 in both the MSKCC and TCGA datasets were identified (Fig. 2a). Surprisingly, although multiple genes were identified as co-mutated in each dataset, none were found in both datasets. DNA damage repair (DDR) pathways reflect the microsatellite instability (MSI) status and are associated with ICI treatment outcomes, but most are not significantly associated with MED12 mutation (Fig. 2b), except for the MMR pathway (*p* < 0.05).

We next assessed the relationship between MED12 mutation and clinical information and previously reported biomarkers. MED12 mutation was independent of age, gender and stage in most datasets (Table 1). Correlation between MED12 mutation and TMB was evaluated and no significant association was observed (*p* > 0.05) in either MSKCC or TCGA dataset (Fig. 2c, ​Analyses in Naiyer2015 and Hira2018 were omitted due to limited MED12-mutant samples (*n* = 3). Cox multivariate regression was performed in MSKCC dataset, and the result indicated that MED12 is also a significant predictive marker, while age and gender were not (Fig. 2d). In addition to the TMB values, MED12 is also independent of IPS (Immunophenoscore) CTLA4/PD1 status (Fig. 2e, *p* > 0.05), or PD-1/PD-L1 gene expression (Fig. 2f). In addition to these biomarkers, BCR and TCR status are also indicators for ICIs response, and MED12 mutation showed no association with BCR/TCR clonality.(Fig. 2g). Immune subtype are also a predictive marker for ICI, which prompted us to investigate their relationships with MED12 mutation. As a result, they are not statistically significant (Fig. 2h). MED12 mutation is an independent predictive biomarker for ICI-treated NSCLC, unaffected by clinical variables or coexisting genomic alterations.

### MED12 mutation was associated with immune related pathways

**Fig. 3:**
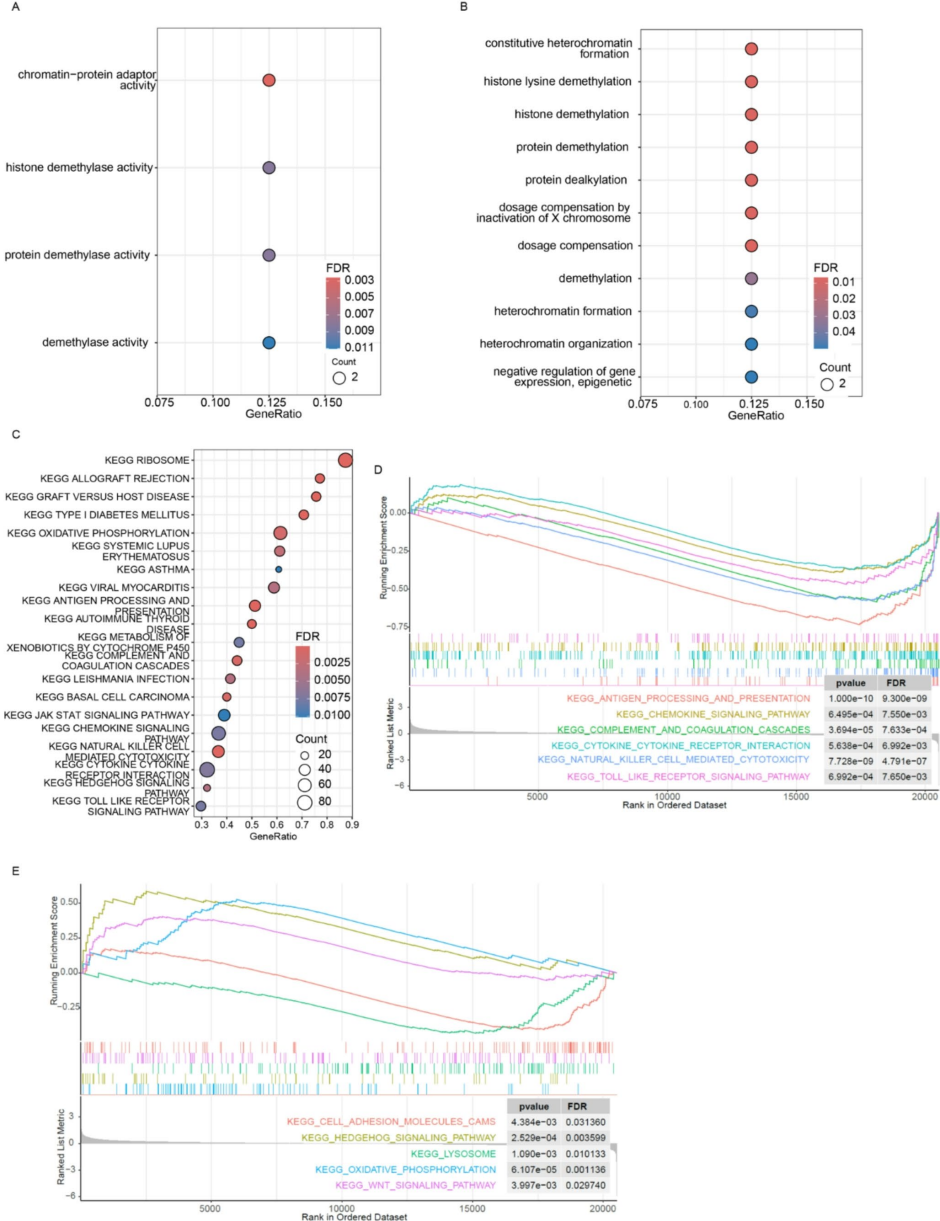
MED12 mutation was associated with immune related pathways​. **a**-**b**, the enrichment of molecular functions and biological processes of differentially expressed genes between MED12-mutant and MED12-wild-type groups; **c**, the Gene Set Enrichment Analysis (GSEA) comparing MED12-mutant and MED12-wild-type groups; **d**, the enrichment of MED12-mutant samples in canonical pathways (all adjusted *p*-values < 0.05); **e**, the enrichment of MED12-mutant samples in immune pathways

To further investigate the impact of MED12 mutation, we identified differentially expressed genes between MED12-mutant and MED12-wild-type groups. Subsequently, enriched Gene Ontology (GO) and KEGG pathways were analyzed. The enriched molecular functions and biological processes included epigenetic regulation, particularly histone modifications (Figs. 3a and b), which are key for transcriptional regulation. Gene Set Enrichment Analysis (GSEA) comparing MED12-mutant versus wild-type groups (Fig. 3c) revealed significant enrichment not only in canonical pathways—such as cell adhesion, JAK-STAT signaling, oxidative phosphorylation, and WNT signaling (Fig. 3d)—but also in immune-related pathways, including antigen processing and presentation, cytokine-cytokine receptor interaction, chemokine signaling, NK cell-mediated cytotoxicity, and TLR pathways (Fig. 3e). Overall, these results indicate that MED12 mutations impact multiple cancer-related biological processes in NSCLC, particularly immune responses and transcriptional regulation.

### MED12 mutation and immune infiltration

**Fig. 4:**
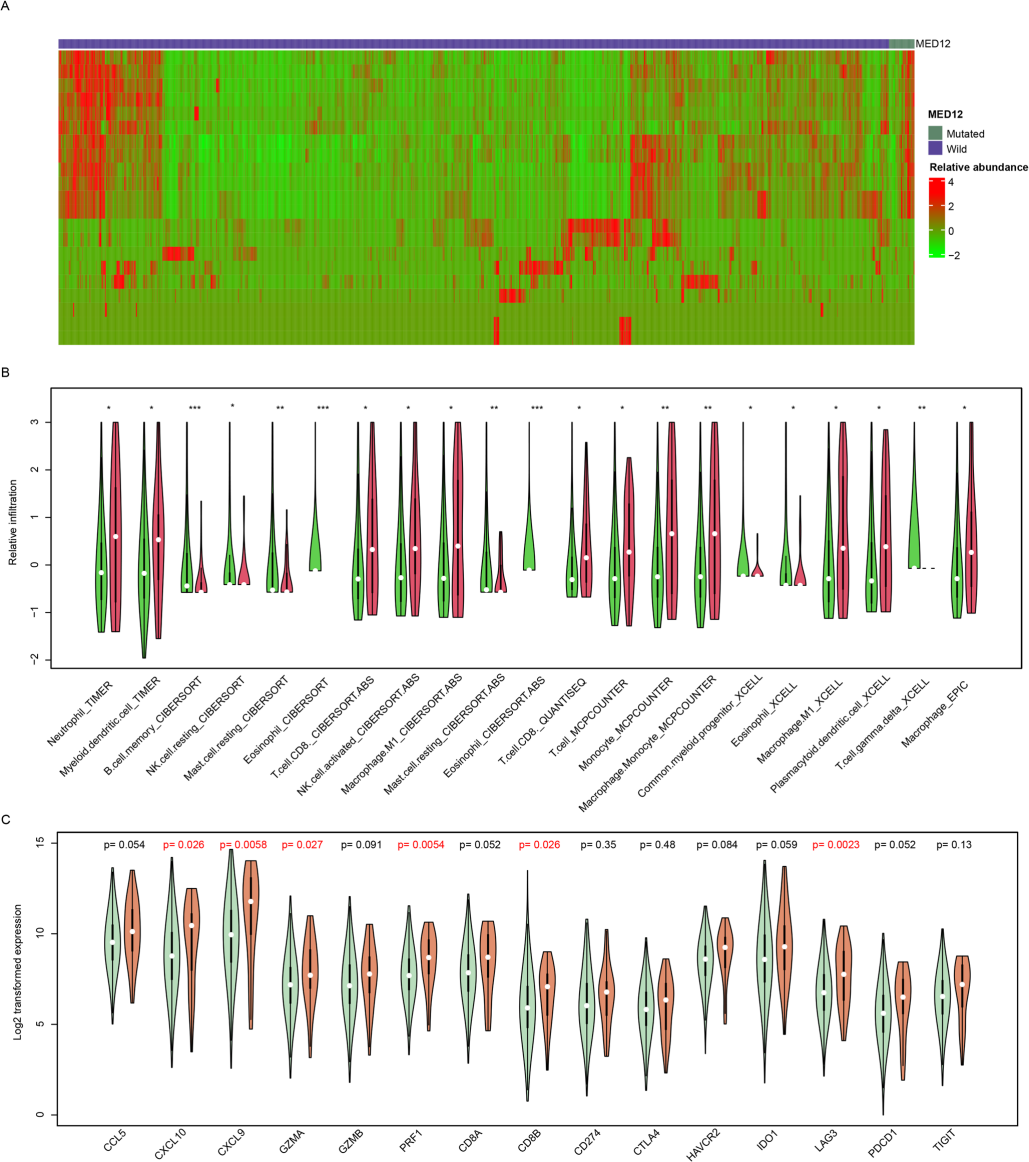
MED12 Mutation and Immune Infiltration​. **a**, the differential infiltration of immune cells between MED12-mutant and MED12-wild-type groups in the TCGA dataset; **b**, the infiltration levels of various immune cells in MED12-mutant and MED12-wild-type groups; *, *p* < 0.05; **, *p* < 0.01; ***, *p* < 0.001. **c**, the expression levels of biomarkers (CXCL10, CXCL9, GZMA, PRF1, CD8B, LAG3) for immune cells in MED12-mutant samples

Since the MED12 mutation status is significantly correlated with immune pathways, we next investigated the relationship between MED12 mutation and immune infiltration levels. The abundances of immune cells were estimated using algorithms including CIBERSORT, XCELL, QUANTISEQ etc. based on the transcriptome of TCGA dataset. As a result, a high proportion of immune cells showed differential infiltration between the MED12-mutant and MED12-wild-type groups (Fig. 4a). Notably, most immune cells were highly infiltrated in the MED12-mutant group, including CD8 + T cells, activated NK cells, M0/M1 macrophages, and neutrophils (Fig. 4b). However, immune cell types including memory B cells, eosinophils, and γδT cells were less abundant in the MED12-mutant group. The expression of biomarkers for immune cells was also evaluated, and it was found that CXCL10, CXCL9, GZMA, PRF1, CD8B, and LAG3 were highly expressed in MED12-mutant samples (Fig. 4c). Collectively, these findings suggest that the MED12-mutant group exhibits an immune-activated phenotype compared to the wild type.

### MED12 suppresses antigen processing gene TAP2 expression

**Fig. 5:**
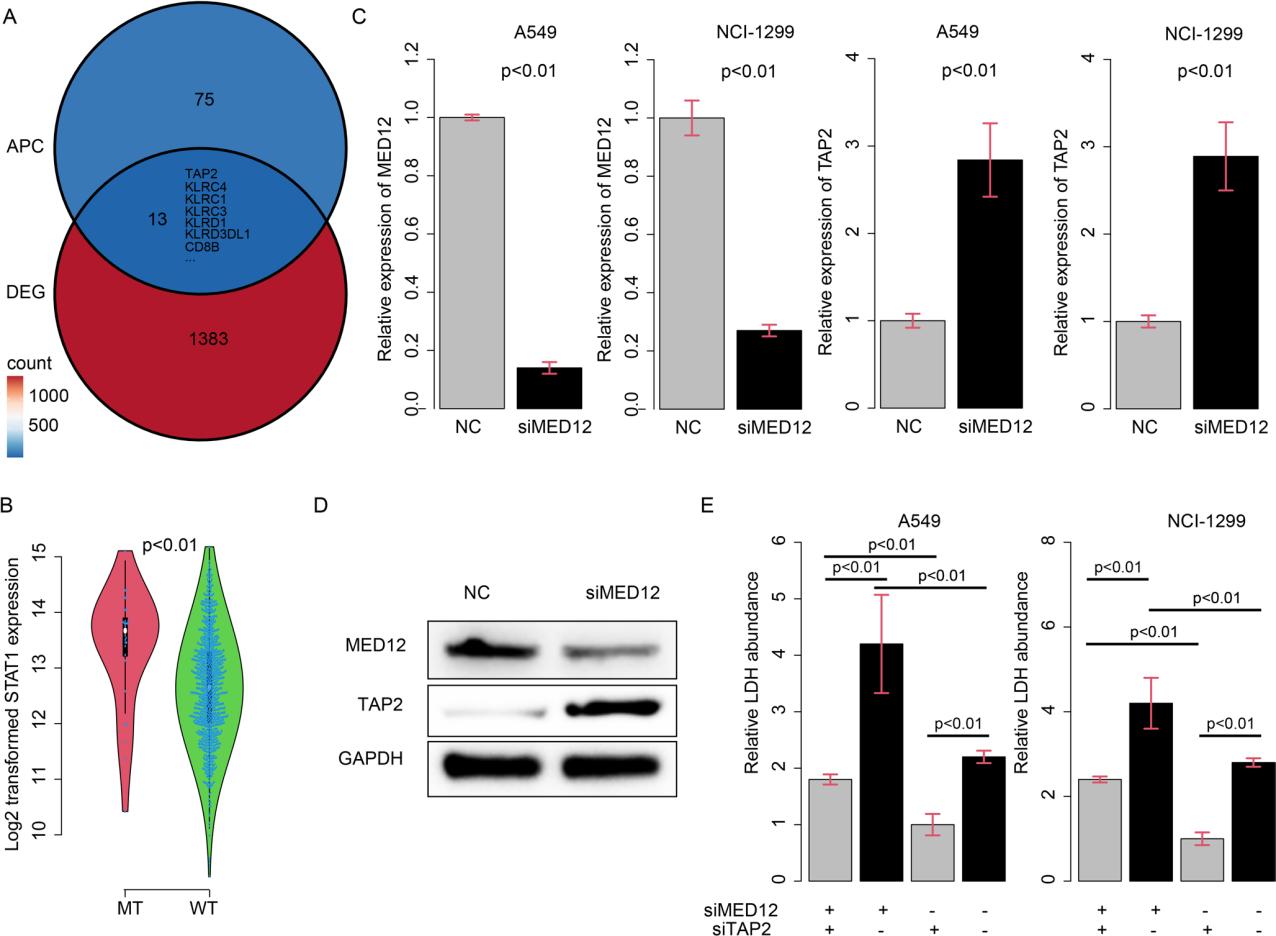
MED12 suppresses antigen processing gene TAP2 expression​. **a**, the identification of genes by intersecting differential genes between MED12 - wildtype/mutated samples (DEG-MED12wm) and antigen processing genes (APGs); **b**, the expression level of TAP2 in MED12-mut and MED12-wild-type samples; **c**, the mRNA level of TAP2 after knocking down MED12 in A549 and NCI-1299 cell lines compared to the control; **d**, MED12 knockding down up-regulated TAP2 protein abundance; **e**, the lactate dehydrogenase (LDH) levels to assay T cell cytotoxicity after knocking down TAP2 and MED12 in cancer cells and co-culturing with CD8 + T cells

As the antigen-processing pathway was significantly enriched and activated immune cells were infiltrated in MED12-mut samples, we proposed that MED12 mutation may affect the gene expression of antigen processing genes (APGs) by regulating transcription initiation as a transcription factor. However, when intersecting differential genes between MED12-wildtype/mutated samples (DEG-MED12wm), with APG and predicted MED12 targets according to TFlink(Liska et al. 2022) database, we obtained two genes, KLRC1 and KIR2DL4 (not shown). Both genes are killer cell receptors expressed in immune cells, while MED12 mutation occurs only in cancer cells. MED12 is unlikely to regulate their transcription initiation in immune cells. Thus, we suspect that MED12 may regulate the expression of transcription factors and subsequently modulate expression of APGs in cancer cells. Thirteen genes were identified by intersecting DEG-MED12wm and APGs (Fig. 5a). Among these genes, TAP2 was noticed due to its role in antigen processing and the T cell activation process reported recently(Ranjan et al. 2025). The other genes are specifically expressed in immune cells, especially killer cells. MED12 mutation significantly enhanced the expression of TAP2 (Fig. 5b). To validate this finding, we knocked down the expression of MED12 in A549 and NCI-1299 cell lines, and found that the mRNA level of TAP2 was significantly enhanced (Fig. 5c), compared to the control, indicating that MED12 suppresses the expression of TAP2. The protein levels were also consistent with this (Fig. 5d). In addition, we knocked down TAP2 and MED12 in cancer cells, and then co-cultured these cancer cells with CD8 + T cells, and quantified lactate dehydrogenase (LDH) levels to assay T cell cytotoxicity, as a consequence of antigen processing and presentation. As expected, TAP2 knockdown reduced while MED12 knockdown enhanced T cell cytotoxicity (Fig. 5e). The knockdown of TAP2 restored the enhanced LDH level caused by MED12 knockdown. Besides, we also mutated the MED12 (S1809R and P636L). In consistent with our hypothesis, the mutation effects of MED12 was similar as MED12 knocking down (Fig. S3), which yielding a restored TAP2 expression and cytocytoxity, compared to the wild type. Taken together, these results indicate that MED12 suppresses antigen processing and T cell cytotoxicity by down-regulating TAP2.

### MED12 suppresses TAP2 and CD8 + T cell cytotoxicity via STAT1/STAT2/IRF1

**Fig. 6:**
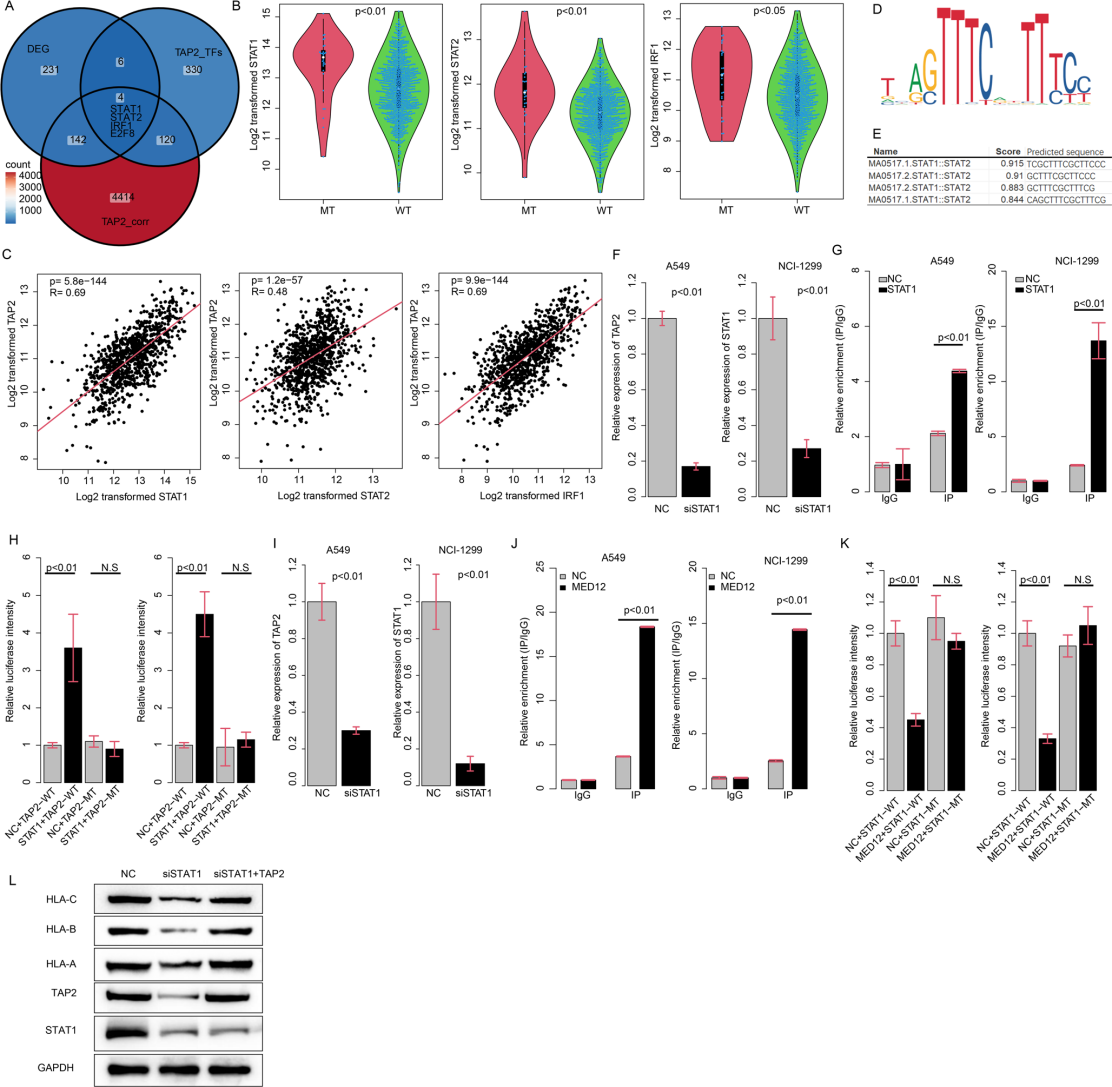
MED12 Suppresses TAP2 and CD8 + T Cell Cytotoxicity via STAT1/STAT2/IRF1​. **A** Identification of four candidate genes (STAT1, STAT2, IRF1, E2F8) via intersection of gene sets; (**B**) Expression of STAT1, STAT2, and IRF1 in MED12-mutant vs. wild-type samples; (**C**) Correlation between STAT1 (R² = 0.69), STAT2 (R² = 0.48), IRF1 (R² = 0.69), and TAP2 expression; (**D**) STAT1/STAT2 binding motifs predicted by the JASPAR database; (**E**) Validated STAT1/STAT2 binding sequence in the TAP2 promoter; (**F**) TAP2 mRNA levels after STAT1 knockdown in NSCLC cell lines (A549, NCI-H1299); (**G**) ChIP-PCR confirming STAT1 enrichment at the TAP2 promoter; (**H**) Luciferase activity of wild-type (WT) vs. mutated (MT) TAP2 promoter; (**I**) ChIP-PCR showing MED12 enrichment at the STAT1 promoter; (**J**) STAT1 mRNA levels after MED12 knockdown; (**K**) Luciferase activity of the STAT1 promoter with/without MED12. N.S., not significant; WT, wild-type; MT, mutant; (**L**) Protein levels of TAP2 and MHC-I (HLA-A/B/C) in STAT1 knockdown and TAP2 overexpression cells. N.S, not significant

Based on the previous hypothesis, we next investigated the transcription factor that regulates the transcription initiation of TAP2 and is regulated by MED12. The TAP2 transcription factors predicted from the TFlink database (TAP2_TFs) and genes whose expression was significantly associated with TAP2 (TAP2_corr) were identified. By intersecting DEG-MED12wm, TAP2_TFs and TAP2_corr, we obtained four genes, named STAT1, STAT2, IRF1 and E2F8 (Fig. 6a). Since IRF1, STAT1 and STAT2 form a transcription regulatory complex, we suspect that MED12 regulates the expression of STAT1/STAT2/IRF1 and thus modulate the expression of TAP2. The MED12 mutation significantly reduced the expression of STAT1, STAT2 and IRF1 (Fig. 6b), and STAT1/STAT2/IRF1 gene expression level was significantly correlated with TAP2, accounting for 69%, 48% and 69% of the TAP2 expression variance, respectively (Fig. 6c). Using JASPAR database, we retrieved the STAT1/STAT2 binding motif (Fig. 6d) and identified the binding sequence (Fig. 6e). Knockdown of STAT1 significantly reduced the expression of TAP2 in A549 and NCI-H1299 cell lines (Fig. 6f). ChIP-PCR revealed that STAT1 was significantly enriched in the promoter region of TAP2 (Fig. 6g). The luciferase reporter assay showed that the wild type promoter of TAP2 increased luciferase intensity, while the mutated promoter did not (Fig. 6h), indicating that the STAT1 complex promotes transcription initiation of TAP2. In addition, we also investigated the role of MED12 in regulating transcription of STAT1. MED12 was enriched in the promoter region of STAT1 according to ChIP-PCR (Fig. 6i). However, knockdown of MED12 significantly enhanced the mRNA level of STAT1 (Fig. 6j), and the promoter of STAT1 resulted in significantly decreased luciferase intensity in the presence of MED12 (Fig. 6k), indicating that MED12’s binding to the STAT1 promoter region repressed its expression. On the other hand, mutation of MED12 showed a similar pattern based on ChIP-PCR and luciferase assay (Fig. S4-S5). In addition, we assayed the protein abundance after knocking down STAT1 and/or overexpression of TAP2 (Fig. 6l). Knocking down of STAT1 significantly reduced the protein abundance of TAP2, HLA-A, HLA-B and HLA-C. The overexpression of TAP2 restored its effect. We also analyzed the effect of IRF1 and STAT2 and found that both STAT2 and IRF1 impact on the transcription of TAP2 (Fig. S5). Knockding down of STAT1/STAT2/IRF1 reduced the expression of TAP2 by 67%, 66% and 62%, respectively. On the other hand, combination of knocking down of STAT1/STAT2/IRF reduced 81% TAP2 expression. We thus suspect that the may act additively, not synergystically. Collectively, MED12 inhibits the transcription of STAT1, and STAT1 promotes the expression of TAP2 as a transcription factor, and ultimately regulate MHC-I genes HLA-A/B/C.

## Discussion

As an emerging strategy for both late-stage and resectable non-small cell lung cancer (NSCLC) treatment(Dunne et al. 2024), immunotherapy has been widely adopted in clinical practice. However, one of the most significant challenges lies in its heterogeneous efficacy across individuals(Meyer et al. 2024). Compared to other targeted therapies such as tyrosine kinase inhibitors (TKIs), the impact of somatic mutations on immunotherapy response remains poorly understood. Although combination therapies occasionally improve outcomes over monotherapy(Mathew et al. 2024), their associated side effects cannot be ignored. Current prognostic models, including machine learning and decision-tree-based approaches(Rakaee et al. 2025; Zhao et al. 2024). face limitations in robustness due to insufficient sample sizes and incomplete mechanistic insights. Recent studies have explored molecular determinants of immunotherapy response: for example, LAMTOR1 was shown to inhibit PD-L1 exosomal secretion, thereby enhancing anti-tumor immunity(Wu et al. 2024).while metabolic reprogramming was linked to immune checkpoint inhibitor (ICI) efficacy(Zheng et al. 2024). Nevertheless, carcinogenesis—a complex process driven by genetic alterations—requires deeper investigation into mutation-specific effects. In this study, through data mining across multiple cohorts (MSKCC, TCGA, Naiyer2015, Hira2018) and a retrospectively collected institutional dataset (*N* = 295), we identified MED12 mutations as predictive biomarkers for improved survival in ICI-treated NSCLC patients. Mechanistically, MED12 represses STAT1/STAT2 transcription via promoter binding (ChIP-PCR validated), subsequently downregulating TAP2 and MHC-I complex (HLA-A/B/C) expression to impair antigen presentation (Fig. 6). These findings not only establish MED12 as a clinically actionable biomarker but also elucidate its role in modulating HLA-mediated immune recognition.

MED12 is a component of the preinitiation complex, which binds to CDK8, MED13, cyclin C and CKD8 to initiate the transcription process. It has been reported that MED12 functions in maintaining cancer cell proliferation, along with BRD4(Sooraj et al. 2022). Additionally, mutations of MED12 in castration-resistant prostate cancer facilitate cell proliferation via the Hedgehog signaling pathway(Duong et al. 2023). Consistent with this, GSEA revealed that samples with MED12 mutations in this study were also enriched in the Hedgehog signaling pathway. Aznar et al. reported that MED12 mutations promote uterine leiomyoma via TDO2(Zuberi et al. 2023). According to reports by Zhou et al., MED12 mutations also predict the ICI response in several types of cancer(Zhou et al. 2022), aligning with our pan-cancer survival analysis. However, the efficiency for each cancer type is still not reported. In this work, we found that MED12 mutation was significantly associated with prolonged survival in ICI-treated NSCLC patients (MSKCC/Naiyer/our own cohorts), but not non-ICI-treated patients (TCGA cohort), indicating that it is a specific biomarker for ICI treatment of NSCLC, but not standard of care or other treatment type. However, warrants further investigation in preclinical models.

MED12 is a core co-transcription factor that mediates the interaction between DNA-binding transcription factor and polymerase II to initiate transcription. Supporting this role, the TFlink database (Liska et al. 2022) predicts over 5,000 MED12 target genes. Thus, we suspect that MED12 may interacts with transcriptional repressors that bind to STAT1/STAT2/IRF1 promoter regions and thereby repressing their expression. Mutation of MED12 may disrupt the interaction between MED12 and these transcription factors, though experimental validation is required. Previous results indicated that MED12 is a critical protein for the formation of CDK8-mediator complex, and the mutation of MED12 dysregulated the function of CDK8/Cyclin C function and was involved in carcinogenesis(Knuesel et al. 2009; Turunen et al. 2014). CDK8 was also reported to regulates the activation of STAT1 and NK cell cytocytoxity in tumor(Putz et al. 2013; Bancerek et al. 2013). Thus, we suspect that MED12 may regulates the STAT1/TAP2/cytocytoxity via CDK8-dependent manner. The mutation of MED12 at different site may contribute to the binding, to different degree. However, this needs further investigation. For example, whether CDK8 is necessary during the the transcription initiation of STAT1.

TAP is a protein complex and member of the ATP-binding cassette (ABC) family, consisting of TAP1 and TAP2(Lehnert et al. 2016). It is necessary for the formation of peptide loading complex (PLC), a structure essential to transport the peptide to MHC-I(Cresswell et al. 2005). The antigen presentation process is crucial for immune cell infiltration and activation(Pishesha et al. 2022). Previous works indicate that STAT1/IRF1 binds to the promoter region of TAP1 and facilitate its transcription(Brucet et al. 2004), and our work extends this mechanism to TAP2. In this study, we also demonstrated that MED12 mutation drive STAT1 dysregulation, thereby altering TAP2 expression. It is noted that MED12 mutation is not significantly associated with overall or progression-free survival in NSCLC patients treated with other regimens except for ICIs. However, multi-cohort validation confirmed that its mutation predicted survival and clinical response of NSCLC treated with ICIs, suggesting that MED12 is a specific biomarker for ICIs, not other treatment methods. This is consistent with our result. MED12 mutation released the antigen presentation suppression status via STAT1/TAP2 axis to facilitate the efficacy of ICIs, but not other treatment regimens.

The correlations between TAP2 and STAT1/IRF1 (R²=0.69) are higher compared to STAT2 (R²=0.48) with expression. While this data suggests STAT1 and IRF1 may play a more prominent role in directly regulating TAP2 compared to STAT2, despite taht our functional model proposes that STAT1, STAT2, and IRF1 act as part of a transcriptional complex regulating TAP2. The core mechanistic chain we established experimentally places STAT1 as the primary direct target of MED12 repression, with STAT1 knockdown alone significantly reducing TAP2 expression and MHC-I presentation. Indeed, consistent with previous results, we suspect that STAT2 and IRF1 are also probably the target of MED12.

In the immune cell infiltration analyses, the macrophages, especially M1 macrophages, were highly infiltrated in MED12-mut samples, according to different immune cell infiltration algorithms. Antigen processing and presentation process promotes immune infiltration and immune activation, and correlating with improved survival. Specifically, M1 macrophages are key mediators of pro-inflammatory responses(Yunna et al. 2020), which is associated with better survival of NSCLC treated with ICIs(Larroquette et al. 2022). In addition, activated NK cells were significantly increased in MED12-mutant samples compared to MED12-wild-type samples. Previous reports revealed that higher NK cell level is associated with CD8 + T cell activation(Hu et al. 2023). In agreement with this, the CD8 + T cells in MED12-mut samples are also significantly higher. Intriguingly, γδ T cells were paradoxically enriched in MED12-wild-type tumors, despite their emerging role as immunotherapy targets(Mensurado et al. 2023).

A significant challenge inherent to investigating MED12 as a biomarker stems from its relatively low mutation frequency. Across the major datasets analyzed in this study (MSKCC: 3.42%, TCGA: 3.31%, our cohort: 3.39%), the proportion of NSCLC patients harboring non-synonymous MED12 mutations was consistently low, typically around 3–4%. This low prevalence directly resulted in a limited number of MED12-mutant samples available for analysis in each individual validation cohort – notably only 3 samples each in the Naiyer2015 dataset, and a small subset (12 mutants in MSKCC, 31 in TCGA) within the larger datasets. While the consistent observation of significantly prolonged survival in MED12-mutant patients across all ICI-treated cohorts (MSKCC, Naiyer2015, and our own) provides compelling convergent evidence, the small absolute number of mutant cases in each separate cohort inevitably impacts the statistical robustness and precision of the survival estimates within those specific datasets. Small sample sizes increase the susceptibility of hazard ratios and p-values to fluctuation and reduce the statistical power to detect potentially smaller, yet clinically relevant, effect sizes or to perform more granular subgroup analyses. This limitation is particularly evident in the pathological response analysis of the Naiyer2015 dataset, where significance was reached despite only 3 mutant samples, highlighting the vulnerability of such analyses to small numbers. The reliance on retrospective data, where comprehensive clinical information can be missing, further compounds this issue. Therefore, while our multi-cohort validation strategy strengthens the overall conclusion that MED12 mutation is associated with improved ICI outcomes, the low mutation frequency underscores the need for cautious interpretation of the magnitude of effect within individual studies and highlights the critical need for validation in larger, prospectively collected NSCLC cohorts specifically designed to assess this biomarker. Our future efforts will aim to accumulate sufficient MED12-mutant cases through multi-center collaborations to confirm the effect size with greater precision and explore potential interactions with other biomarkers in this subset.

Limitations of this study are as follows. This is a retrospective study; MSKCC and other datasets lack of critical endpoints (durable clinical benefit, objective response rate abd progression-free survival information), which limited the persuasiveness of the results. Another limitation is small sample size of MED12-mutant cases, due to the low mutation frequency of this gene. Thirdly, the predictive value of MED12 mutations needs further validation before clinical use. Fourthly, whether the interaction of MED12 and STAT1 promoter was in CDK8/MED13 dependent-manner is needs further validation. Lastly, functional studies relied on MED12 knockdown rather than mutation modeling. Most MED12 mutations observed were missense variants, classically associated with loss-of-function (LOF); however, while LOF often phenocopies gene knockdown, mechanistic differences may exist. Future work will employ CRISPR-Cas9-mediated introduction of patient-derived MED12 mutations to dissect their functional and therapeutic impacts.

## Conclusions

Mechanistically, MED12 affect the expression of TAP via STAT1 to regulate the antigen presentation process and CD8 + T cell cytotoxicity of NSCLC cell lines. Clinically, MED12 mutation predict the clinical outcome of immunotherapy treated NSCLC.

## Supplementary Information

Below is the link to the electronic supplementary material.

Supplementary Material 1

Supplementary Material 2

Supplementary Material 3

Supplementary Material 4

Supplementary Material 5

Supplementary Material 6

Supplementary Material 7

## Data Availability

The data used in this work were downloaded from databases, and the accession were provided in the main text. The other data is available upon reasonable request.

## Abbreviations

MED12: Mediator Complex Subunit 12
NSCLC: Non-small cell lung carcinoma
NK: Nature killer
TCGA: The Cancer Genome Atlas
TCR: T cell receptor
BCR: B cell receptor
STAT1: Signal Transducer And Activator Of Transcription 1
STAT2: Signal Transducer And Activator Of Transcription 2
IRF1: Interferon Regulatory Factor 1
ICIs: Immune checkpoint inhibitors
DDR: DNA Demage Repair
GSEA: Gene Set Enrichment Analyses
LDH: Lactate Dehydrogenase
TAP2: Transporter 2, ATP Binding Cassette Subfamily B Member
APG: Antigen processing genes
