# Supplementary figures and images for: MED12-STAT1-TAP2 axis regulates CD8 + T cell cytotoxicity and mediates immunotherapy outcome in non-small cell lung cancer
Source: Funct Integr Genomics. 2025 Sep 1;25(1):182. doi: 10.1007/s10142-025-01690-2 (PMC12402025; doi:10.1007/s10142-025-01690-2)

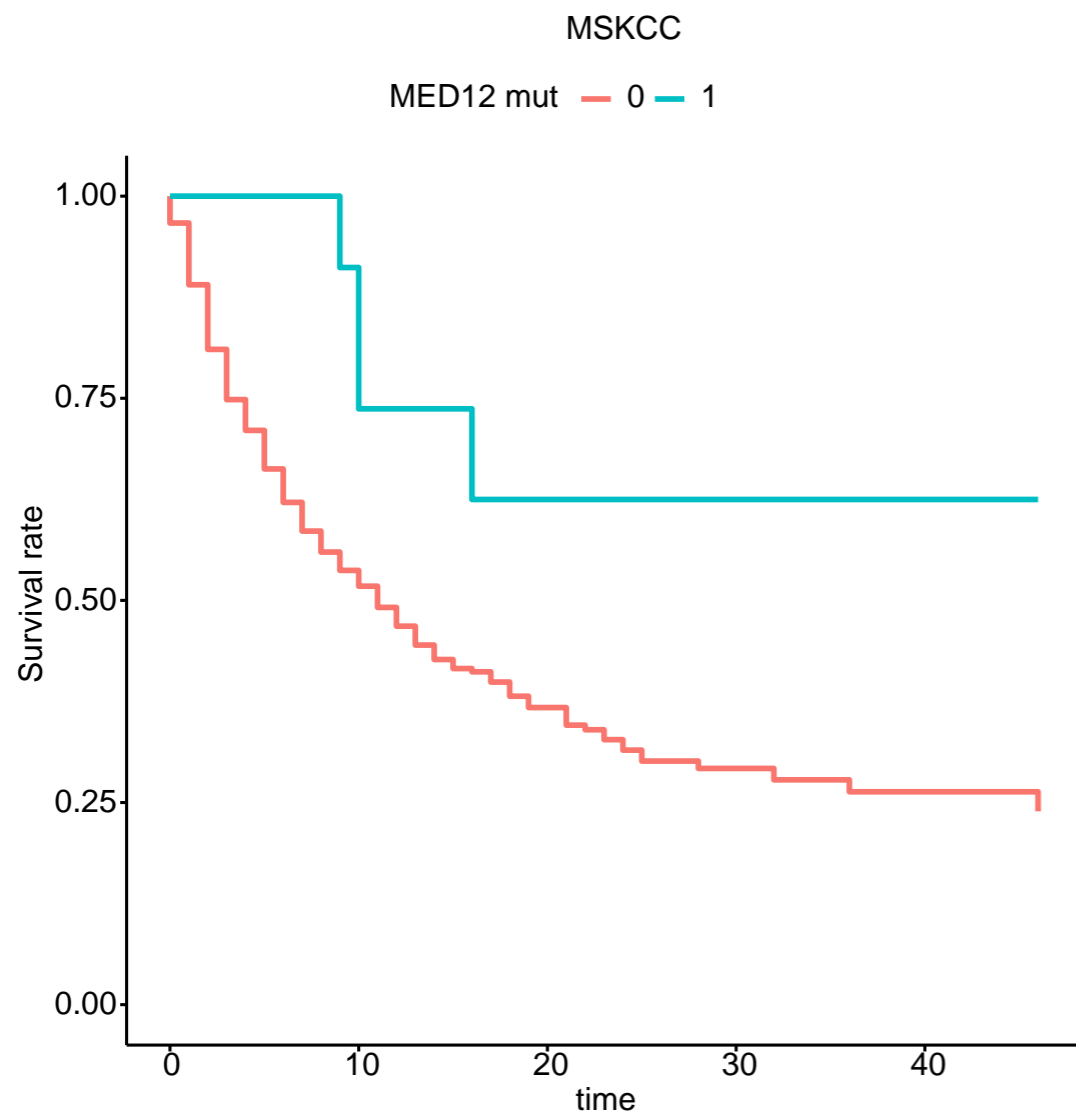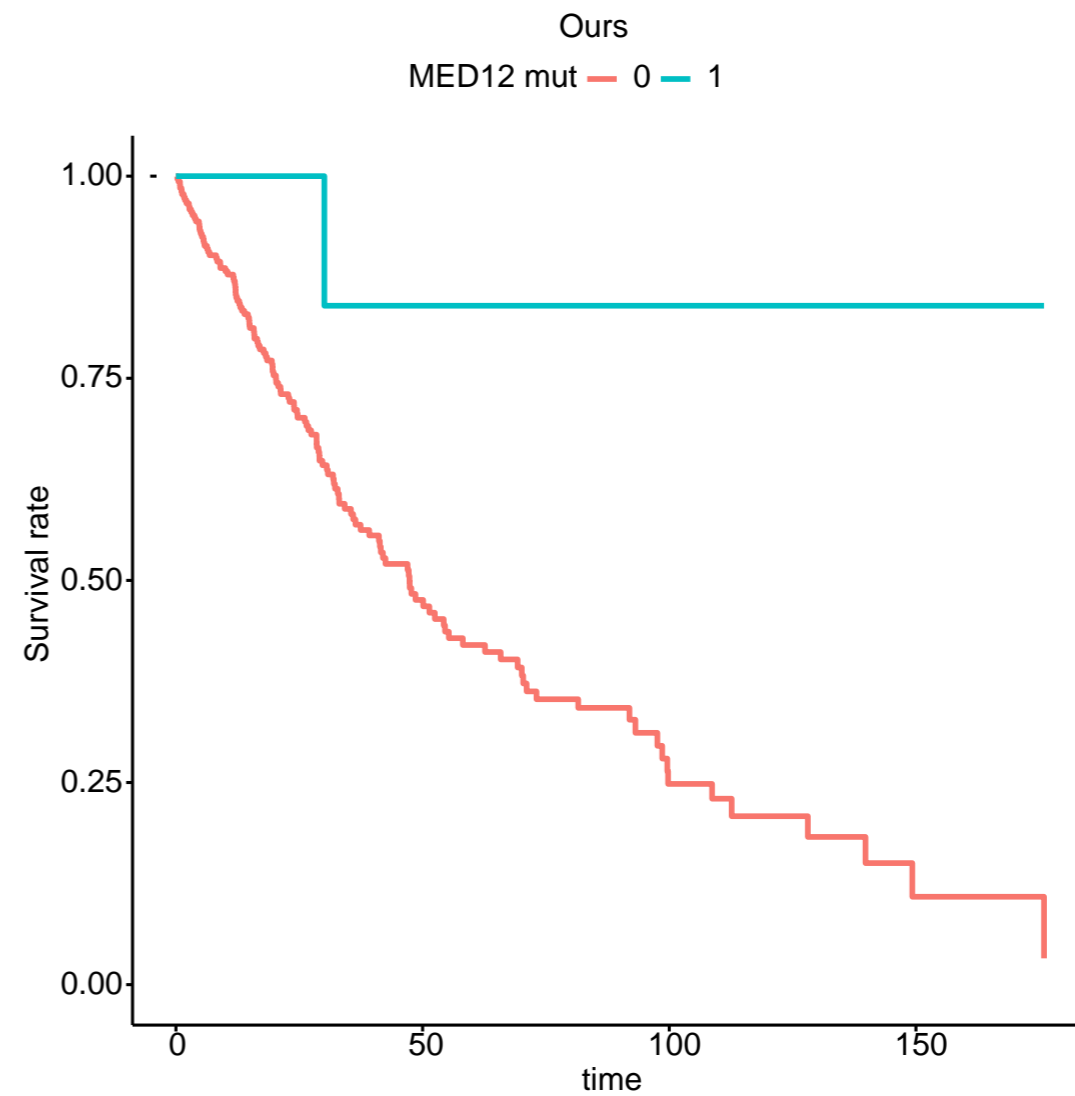

Supplement: Supplementary file 1 — Supplementary Material 1 [file 10142_2025_1690_MOESM1_ESM.pdf]

A

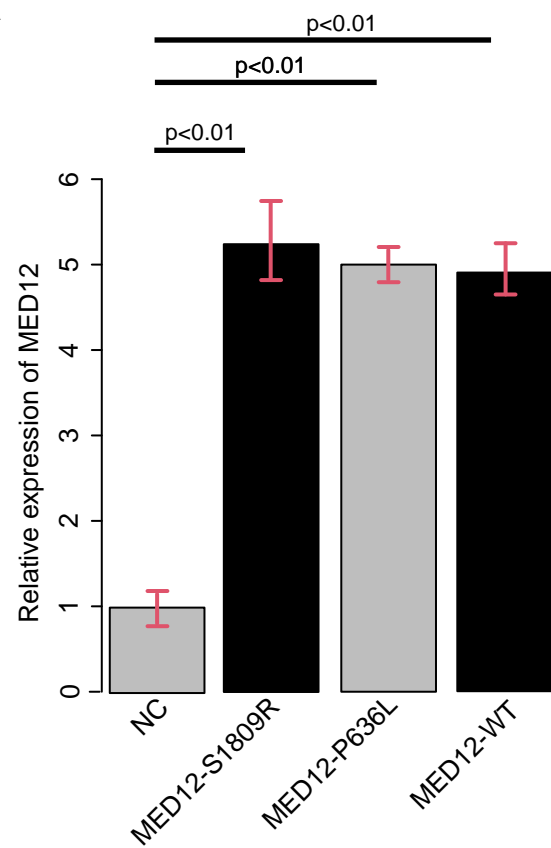

B

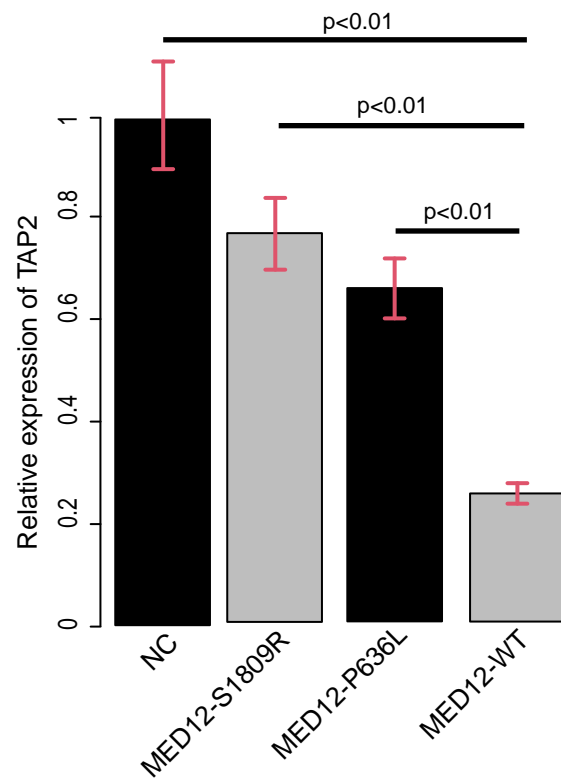

C

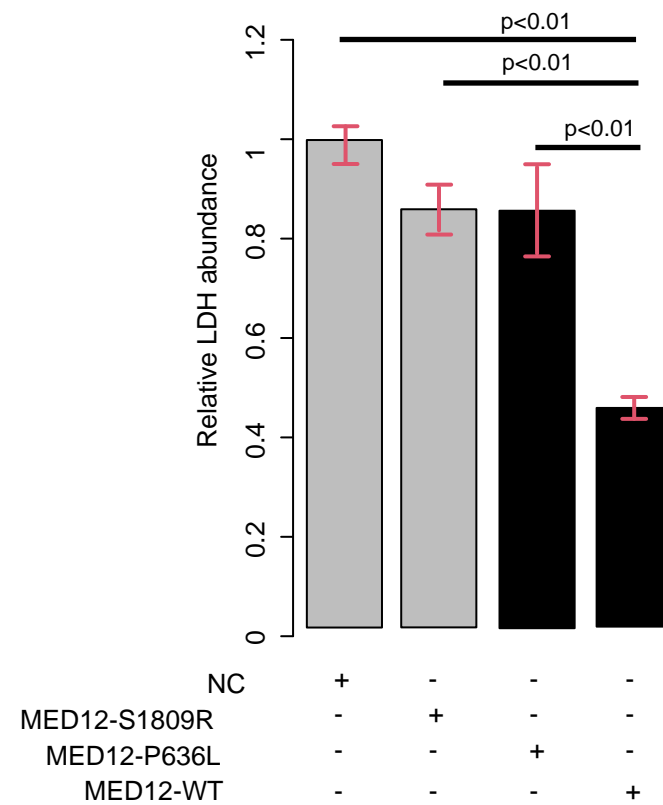

Supplement: Supplementary file 2 — Supplementary Material 2 [file 10142_2025_1690_MOESM2_ESM.pdf]

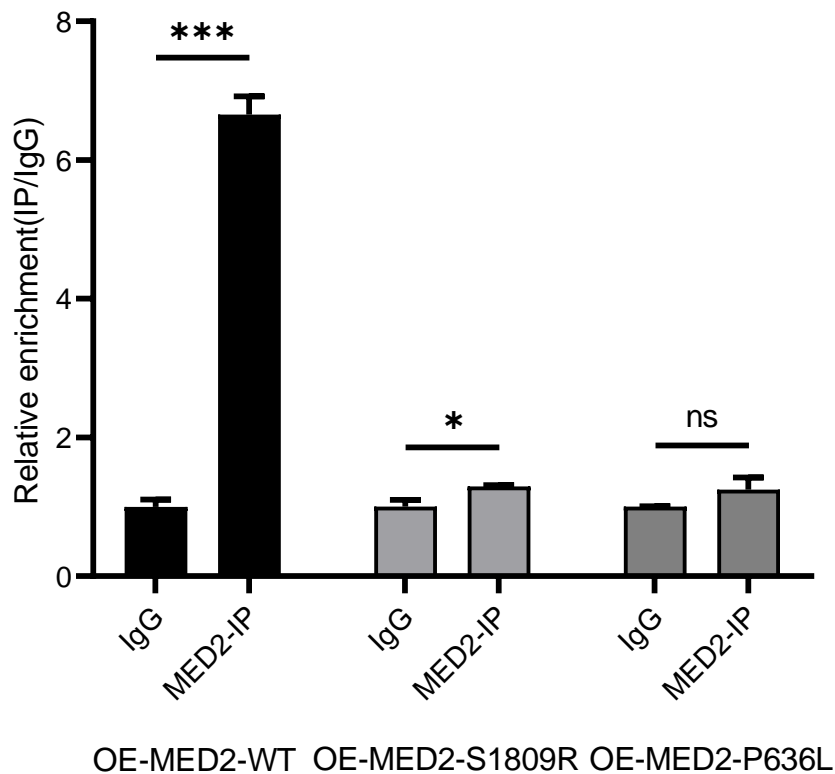

Supplement: Supplementary file 3 — Supplementary Material 3 [file 10142_2025_1690_MOESM3_ESM.pdf]

Relative expression of  
TAP2 mRNA

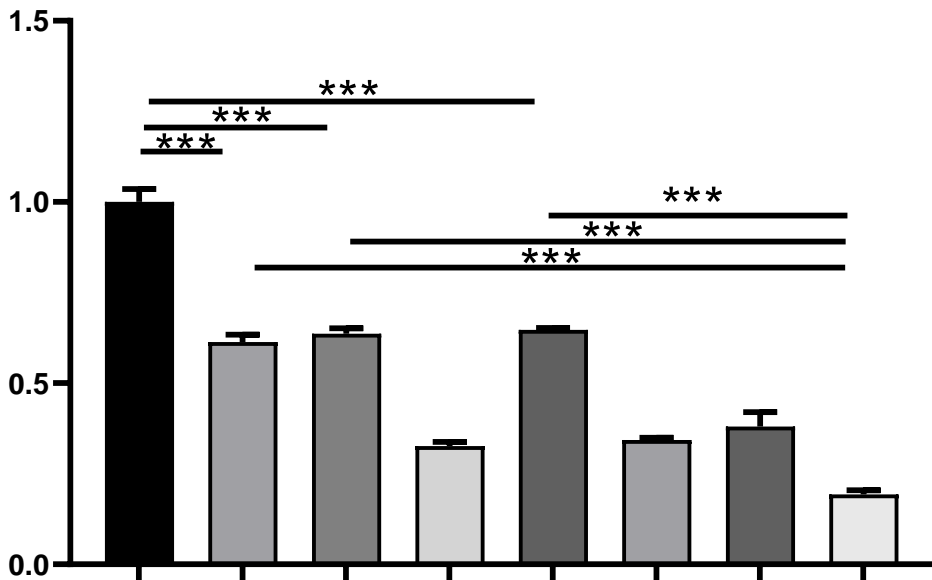

siSTAT1

- - - - + + + +

siSTAT2

- - + + - - + +

siIRF1

- + - + - + - +

Supplement: Supplementary file 4 — Supplementary Material 4 [file 10142_2025_1690_MOESM4_ESM.pdf]

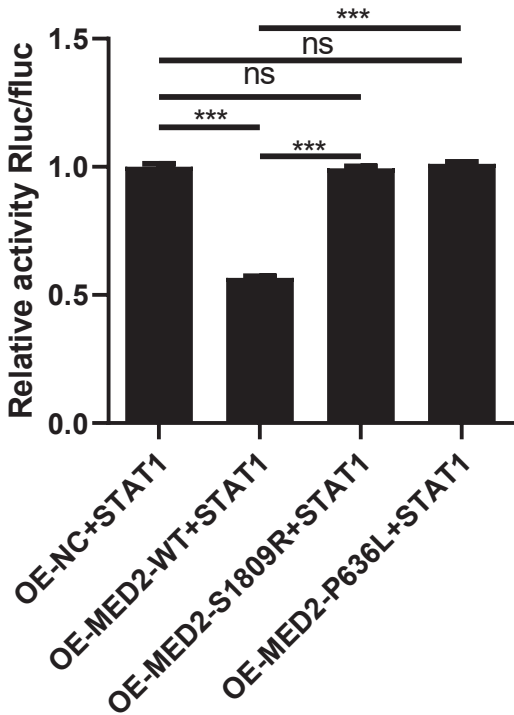

Supplement: Supplementary file 5 — Supplementary Material 5 [file 10142_2025_1690_MOESM5_ESM.pdf]

A

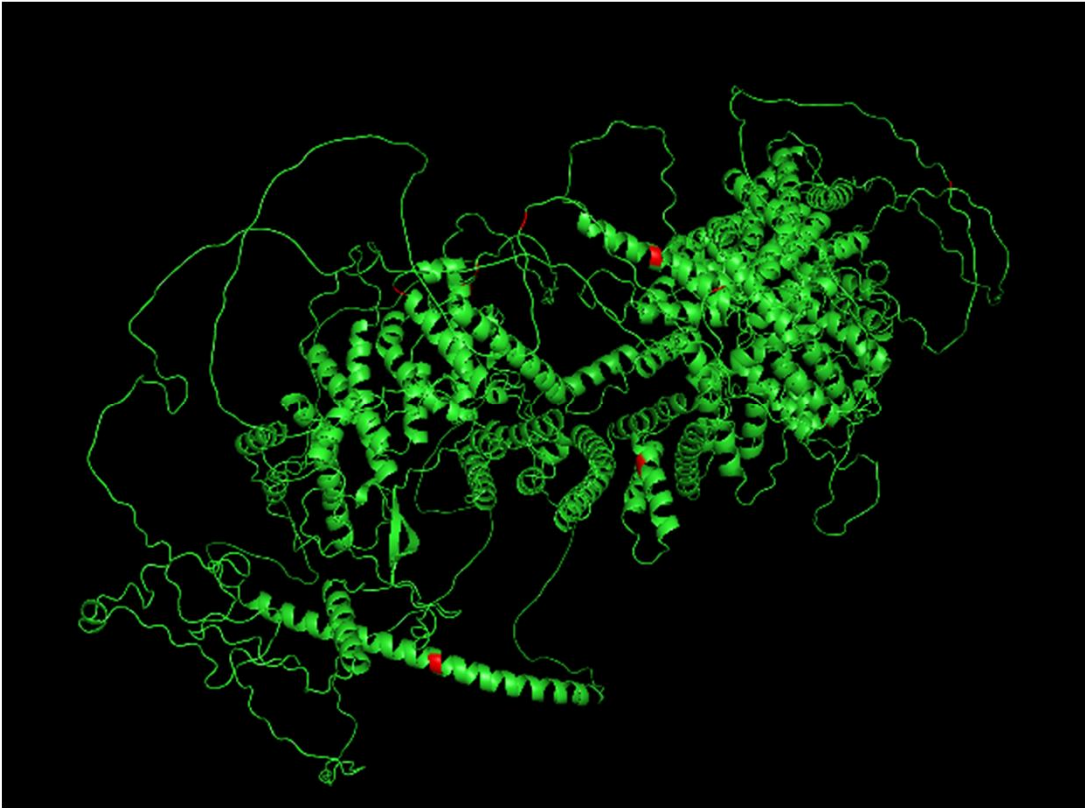

B

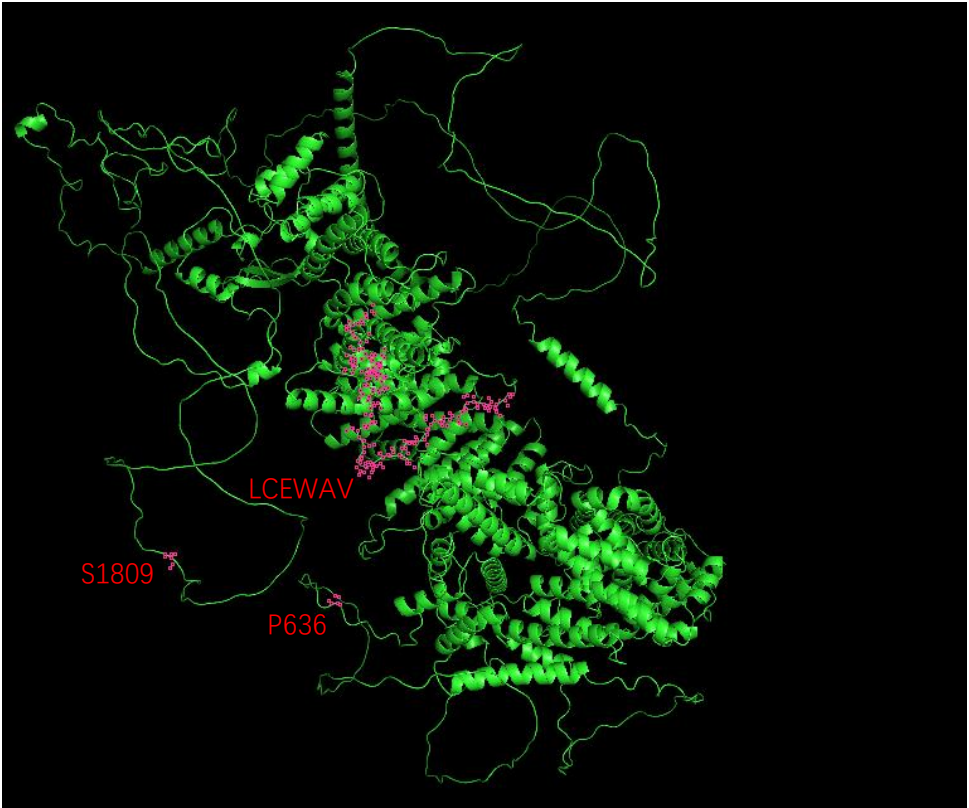

Supplement: Supplementary file 7 — Supplementary Material 7 [file 10142_2025_1690_MOESM7_ESM.pdf]
